# Assessing the reliability and validity of comprehensive score for financial toxicity (COST) among radiation oncology patients in India: a cross-sectional pilot study

**DOI:** 10.3332/ecancer.2021.1219

**Published:** 2021-04-09

**Authors:** Mukhtar Ahmad Dar, Richa Chauhan, Krishna Kumar Sharma, Vinita Trivedi, Sameer Dhingra, Krishna Murti

**Affiliations:** 1 Department of Pharmacy Practice, National Institute of Pharmaceutical Education and Research (NIPER), Hajipur 844102, Bihar, India; 2 Department of Radiotherapy, Mahavir Cancer Sansthan and Research Centre (MCSRC), Patna 801505, Bihar, India

**Keywords:** financial toxicity, COST, reliability, exploratory factor analysis

## Abstract

**Background:**

Besides physical toxicity, cancer care imposes significant financial distress referred to as financial toxicity (FT). FT has become a growing concern among cancer patients. Researchers have associated FT among cancer patients with clinical outcomes like mortality, poor quality of life and non-adherence. Currently, no reliable tools are available for assessing FT among cancer patients in India. The aim of this pilot study was to test the reliability and validity of the Comprehensive Score for Financial Toxicity (COST) questionnaire among patients undergoing radiotherapy in India.

**Material and methods:**

This cross-sectional pilot study was conducted among head and neck cancer patients on follow-up in radiation oncology department. The reliability of COST measure was assessed using Cronbach’s α. The underlying construct of COST was verified by Exploratory Factor Analysis (EFA). EFA was performed using parallel analysis technique.

**Results:**

Based on inclusion and exclusion criteria, the COST questionnaire was administered to 29 patients using the interview method after written informed consent. The COST measure demonstrated excellent reliability with Cronbach’s α of 0.92. A Kaiser–Meyer–Olkin of 0.87 verified the sample adequacy and a *p*-value of ˂0.001on Bartlett’s sphericity test indicated that the strength of the correlation between 11 COST items was good to perform the EFA. Parallel analysis technique identified one factor on scree plot with eigenvalue of 6.21 explaining 56.5% of the variance by non-rotated solution. All the factor loadings in one factor model were ˃0.3 (range 0.35–0.97). The factor loadings indicated that the underlying construct can be considered as one factor domain as intended by the original COST development study. However, Chi-square goodness of fit test revealed the one factor model did not adequately depict the data. However, the results were consistent with the construct obtained in the original scale development study.

**Conclusion:**

This pilot study demonstrated excellent reliability of COST for measuring FT among radiation oncology patients. Further studies are warranted to study the clinical implications of FT in the Indian population for making better strategies and policies to ease the financial burden on cancer patients.

## Introduction

The burden of cancer is increasing at an alarming rate in India. GLOBOCAN 2018 has estimated over 1.1 million incident cancer cases and 0.78 million cancer deaths in India in 2018 [1]. In addition to the physical toxicity, cancer care imposes a significant financial burden on patients. With the development of new drugs, imaging, radiation and surgical techniques, the cost of cancer care has escalated in past few decades. Oncology patients often suffer the economic consequences of cancer treatment including high out-of-pocket (OOP) expenses (direct and indirect costs) and loss of income. The adverse effects of financial hardships experienced by cancer patients because of cancer care are termed as financial toxicity (FT) [2]. Zafar *et al* [2] concluded that FT is analogous to physical toxicity among cancer patients.

A systematic review by Altice *et al* [3] reported that 47%–49% of cancer survivors experienced some form of the financial burden and 12%–62% of the patients reported being in debt because of cancer treatment. Researchers have linked FT with several clinically relevant outcomes like health-related quality of life (HRQOL) indicating negative effects of FT on HRQOL [4, 5]. Ramsey *et al* [6, 7] have reported association of FT with early mortality and greater risk for bankruptcy. While several other studies have reported association of FT with treatment compliance [8], medication non-adherence [9, 10] and forgoing treatment [11].

Studies among Indian cancer patients have reported catastrophic expenditures among 76.5% of the patients [12]. In India, 40% of cancer patients use borrowings, sale of assets as coping strategies to pay for cancer treatment [13]. A study conducted at All India Institute of Medical Sciences (AIIMS), New Delhi reported that about 59% of the cancer care was spent on transportation, food and lodging during radiotherapy (RT) treatment [14]. An OOP expenses study conducted in north India reported the total mean expenditure was ten times the per capita income and indirect costs were significantly higher than direct costs [15].

India is a lower-middle income country with large population and extremely low health insurance coverage. Majority of the medical expenses are OOP expenses. Currently, there are no validated tools for assessment of subjective FT among cancer patients in Indian healthcare system. To the authors’ knowledge, this is the first study in India assessing subjective FT and the authors aim to test the reliability and validity of Comprehensive Score for Financial Toxicity (COST) for measuring FT among radiation oncology patients.

## Materials and methods

### Study design and participants

This cross-sectional pilot study was conducted at Mahavir Cancer Sansthan and Research Centre (MCSRC), Patna, India. The study was approved by Institutional Ethics Committee. Patients aged ≥18 years with a diagnosis of head and neck cancer who have completed the radiation therapy either as stand-alone or part of multimodal treatment regimen were eligible for participation in this study. Those who were not able to understand Hindi or English language or unable to give informed consent were excluded. Consecutive patients attending radiation oncology out-patient department (OPD) at MCSRC were approached to participate in this study. Patients willing to give informed consent were enrolled for participation in this study. All the financial burden related questions were directed towards radiation therapy only. Based on the literature search, a sample size of 30 patients was selected for this study.

### Data collection

Socio-demographic and clinical characteristics were collected from the patient’s record file in a pre-designed case record form. Responses to COST items were recorded via face-to-face interview during follow-up OPD visits. The questions were properly explained one by one to the participants in a language they understand in presence of a family member/caregiver and the responses were recorded accordingly.

### COST administration and scoring guidelines

The COST-Functional Assessment of Chronic illness Therapy (FACIT) was developed by De Souza and colleagues as a patient reported outcome measure for assessment of FT among cancer patients in the USA. The COST development study reported an excellent internal consistency (Cronbach’s α of 0.92), test-retest reliability and correlation with HRQOL [16].

The COST measure has already been validated for assessment of FT in different cancer care settings across the USA including advanced solid tumours [17], radiation oncology [18], gynaecologic oncology [19], multiple myeloma [20], lung cancer [21] and surgery [22]. COST measure has also been translated and adapted for use in different healthcare systems by other countries including Japan [23], Italy [24] and Brazil [25].

A proper permission and licensing agreement was provided by the original developer via FACIT.org to use COST-FACIT (Version 2) in this study. Although COST is a patient reported outcome measure designed for self-administration, however, FACIT administration and scoring guidelines allow for the face-to-face or telephonic interview-based administration [26].

**Supplementary Figure 1.**
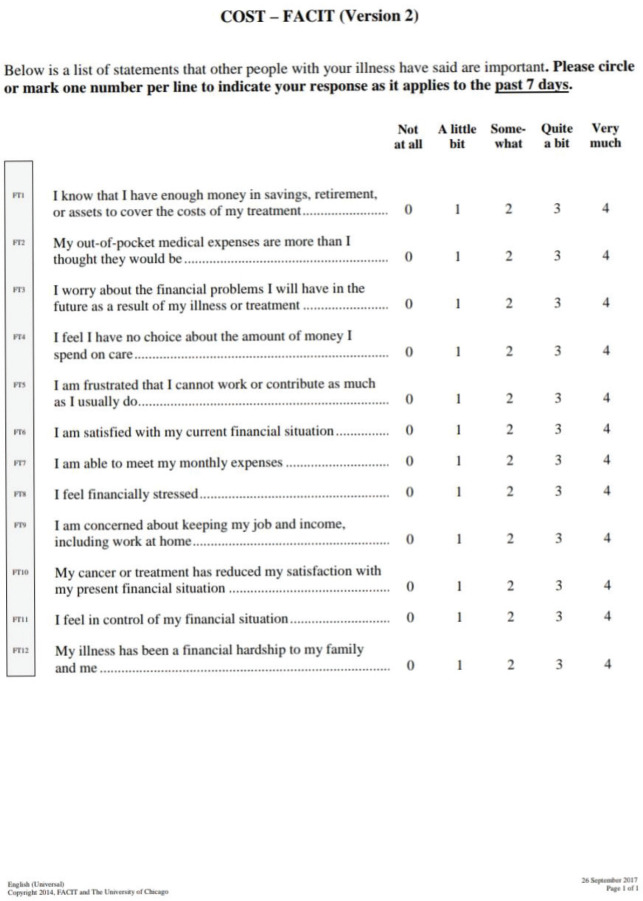
COST-FACIT-Version 2.

The original COST-FACIT (Version 1) was developed on a 5-point Likert scale (0–4) with 11 items. The COST-FACIT (Version 2) is a measure of 12 items (Supplementary Figure 1). The 12 items on COST have been officially coded sequentially from FT1 to FT12 and in this study COST items are presented as codes only. According to FACIT scoring guidelines, the additional item – FT12 is a summary item and does not add to the total score of the questionnaire, i.e. item 12 must be excluded. Items 2, 3, 4, 5, 8, 9 and 10 are reverse scored. The responses vary from 0 (not at all), 1 (a little bit), 2 (somewhat), 3 (quite a bit) and 4 (very much). The total cost score ranges from 0 to 44 and lower score corresponds to higher FT. The total COST score has been subdivided into four groups indicating different grades of FT. Thus, COST score ≥26 indicates no impact (Grade 0), 14–25 means mild impact (Grade 1), 1–13 means moderate impact (Grade 2) and a score of 0 means high impact (Grade 3).

### Statistical analysis

The individual responses to the COST questionnaire were entered into SPSS (Version 20) for analysis. The reliability or internal consistency of COST indicating the degree to which the individual items comprising the scale measure the underlying construct was assessed by the Cronbach’s α. Coefficients ˃0.9 were considered excellent. Spearman’s correlation was performed to demonstrate the correlation between the scale items. Kaiser–Meyer–Olkin (KMO) test to measure sampling adequacy, Bartlett’s sphericity test was performed to test the significance of all the correlations within the correlation matrix. Exploratory Factor Analysis (EFA) was performed for factor identification based on the KMO and Bartlett’s sphericity test [27, 28].

## Results

Based on inclusion and exclusion criteria, the COST questionnaire was administered to 29 patients via interview method for this pilot study. A total of 30 head and neck cancer patients on follow-up after RT were approached for participation in this study. One patient was excluded as the patient was unable to give consent and was not accompanied by a family member/caretaker. The socio-demographic and clinical characteristics of the participants are presented in Table 1. The mean age of the participants was 49.5 ± 16.8 (range 20–74) and 82.8% were male.

### Reliability analysis

The 11 items for COST measure had a Cronbach’s α of 0·92 (95% confidence interval) indicating excellent reliability. No increase in Cronbach’s α values was observed if any of the individual items were deleted from the scale as indicated in Table 2.

Spearman’s correlation test was performed for all the items in the COST as shown in Table 3. Cohen's standard was used to evaluate the strength of the relationships, where coefficients between 0.10 and 0.29 represent a small effect size, coefficients between 0.30 and 0.49 represent a moderate effect size and coefficients above 0.50 indicate a large effect size [29].

A significant positive correlation was observed between FT1 and FT3 (*r*_s_ = 0.68, *p* < 0.001, 95% CI (0.41, 0.84)). The correlation coefficient between FT1 and FT3 was 0.68, indicating a large effect size. This correlation indicates that as FT1 increases, FT3 tends to increase. A similar large effect size was observed in 30 other correlation combinations as depicted by *r*_s_ values in Table 3. A significant positive correlation was observed between four correlation combinations indicating a moderate effect size. A total of 70/110 correlation combinations were significant based on *p-*value indicating 63.6% significant correlation.

### Exploratory factor analysis

**Figure 1.**
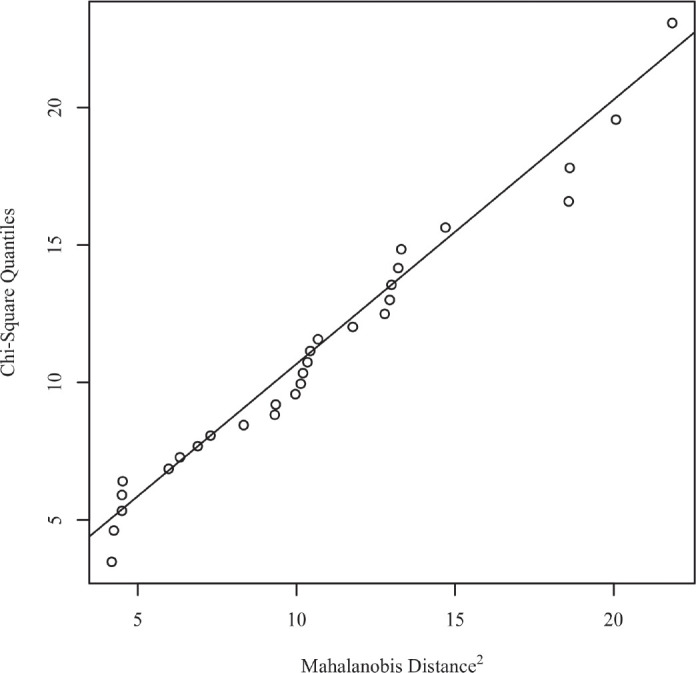
Mahalanobis distance scatterplot testing multivariate normality.

Normality of the data was verified by multivariate normality. The squared Mahalanobis distances were calculated for the data and plotted against the quantiles of a Chi-square distribution. In the scatterplot, the solid line represents the theoretical quantiles of a normal distribution. Normality can be assumed if the points form a relatively straight line. The scatterplot for normality is presented in Figure 1.

EFA was performed after KMO and Bartlett’s test verified the sample adequacy and significant correlation between 11 COST items. Results of KMO and Bartlett’s test are presented in Table 4. A KMO ˂0.8 verifies the sample adequacy and *p*-value of ˂0.001 in Bartlett’s test indicates the strength of the relationship between COST items was good and it is possible to perform EFA on this data.

**Figure 2.**
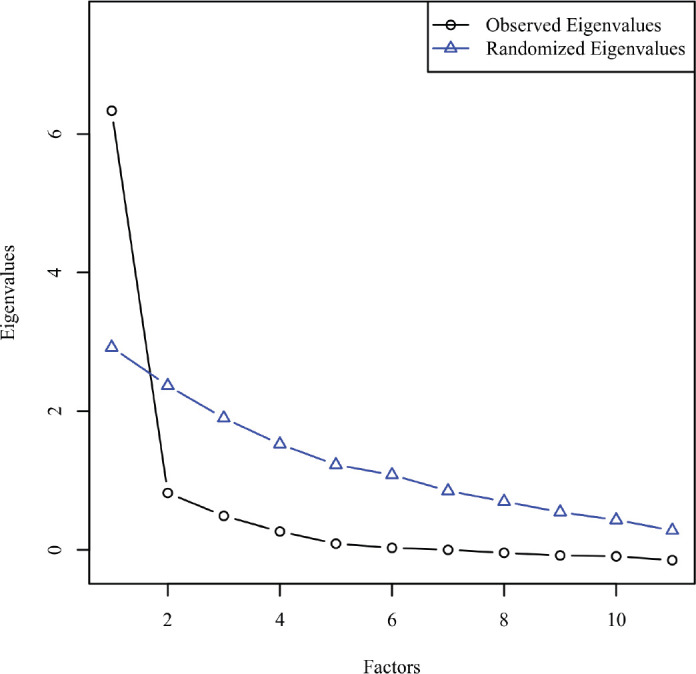
Scree plot comparing observed and random eigenvalues for parallel analysis.

Parallel analysis was chosen for electing number of factors to retain. For this selection method, uncorrelated normal variables are randomly generated that parallel the data in the number of variables and sample size. Next, the observed eigenvalues were extracted from the correlation matrix with the diagonal of the matrix being replaced by each variable's squared multiple correlations to estimate each variable's communality. These observed eigenvalues are then compared to the eigenvalues of the randomly generated variables. The actual eigenvalues that have a higher value than its randomly generated counterpart are retained for interpretation [30]. Figure 2 shows the scree plot comparing the observed and random eigenvalues. Scree plot shows that there was only one factor that had a greater eigenvalue than its randomly generated counterpart. As a result, one factor with eigenvalue of 6.21 explaining 56.5% of the variance by non-rotated solution was identified in factor analysis.

The factor loadings were interpreted by taking the absolute value of each loading and implementing the criterion by Tabachnick *et al* recommending that values ˃0.32 should be the minimum threshold used to identify significant factor loadings [31]. Six items (FT1, FT3, FT6, FT8, FT9, FT10 and FT11) on COST measure showed excellent loadings for Factor 1, one item (FT7) showed very good loading for Factor 1, one item (FT5) had good loading for Factor 1 and two items (FT2, FT3) showed only acceptable factor loadings of ˃0.3 for Factor 1 as shown in Table 5. A Chi-square goodness of fit test was conducted to determine if the one-factor model fits the data perfectly based on an alpha value of 0.05, χ^2^(44) = 60.82, *p* = 0.047 indicating that the one-factor model did not adequately depict the data due to low sample size.

*Financial toxicity measures*

**Figure 3.**
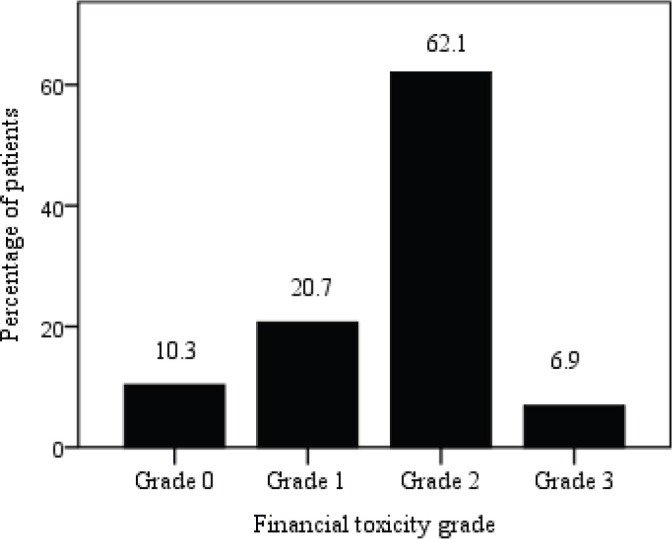
Patient distribution by FT grade; COST score ≥26 (Grade 0), 14–25 (Grade 1), 1–13 (Grade 2) and 0 (Grade 3).

The mean COST score for 29 patients included in this study was 10.8 ± 9·6 (range 0–33) and 62% patients suffered moderate impact (Grade 2) while 7% experienced high impact (Grade 3) FT. Figure 3 shows the distribution of patients by grading COST score using COST-FACIT scoring guidelines.

Patient-oncologist cost communication behaviour and cost coping strategies were also assessed during interview process (Table 6). Almost 90% of the patients discussed their financial situation and affordability of radiation therapy and associated tests to their radiation oncologist. Multiple strategies were employed by patients to pay for cancer care, 86% applied for government financial aid available for people below poverty line, 55% used their savings and 69% borrowed money from social nets. Due to cancer, 62% reported stopped working and about 21% reported reduction in working hours.

## Discussion

A total of 29 head and neck cancer patients attending radiation oncology OPD on follow-up were included in this study. Almost 83% of the study participants were male and 62% cases were cancers of lip and oral cavity. According to GLOBOCAN-2018, lip-oral cancers are second leading cause of incidence (10.4%) and mortality (9.3%) among cancer patients in India following breast cancer. Lip-oral cancers are the leading cause of incidence (16.1%) and mortality (12.3%) among males in India [32].

All the patients (100%) belonged to rural residences, 34% of the participants were not educated, 48% had only primary level education, 34.3% were manual labourers, 10% were unemployed, 13·8% were employed and others were farmers and housewives. Also 58.6% reported less than Rs. 50,000 annual household income, while 38% had 0.5–1 lakh annual income. 93% participants had no knowledge of health insurance and 62% reported stopped working due to cancer. The sociodemographic data in this study correlates with the data from The Ministry of Rural Development, Government of India: Socio Economic and Caste Census (SECC) 2011 [33]. According to SECC, Bihar state has 89% rural households, 44% illiteracy, 20% below primary level education, 54.8% landless households earning by manual labour, 3.8% households with salaried job income and 71% households having less than Rs. 5000 monthly income [33].

The results of our pilot study show that internal consistency of COST measure was excellent with a Cronbach’s α of 0.92 and no increase in Cronbach’s α values was observed if any individual items were deleted, corroborating with the results of the original COST development study (Cronbach’s α = 0.92) [16]. The Cronbach’s α coefficient was higher in this study as compared to validation studies in Japanese, Italian and Brazilian studies which reported a Cronbach’s α of 0.87, 0.83 and 0.82, respectively [23–25].

Spearman’s correlation test for all the items in the COST measure demonstrated that the items have a good correlation with each other. A significant positive correlation with large effect size was observed in 31 correlation combinations as depicted by *r*_s_ values. A total of 70/110 correlation combinations were significant based on *p-*value indicating 63.6% significant correlation. These results were lower as compared to Brazilian version of COST which showed 78.2% significant correlations on Spearman’s correlation test [25]. However, this study showed higher number of large effect size correlations combinations (31) in all items as compared to Brazilian version which reported only three stronger correlation combinations in all items.

KMO and Bartlett’s test demonstrated the sample adequacy and strength of the relationship between COST items were good supporting the factorial analysis. Again, these results were comparable with the original COST development study which reported a KMO of 0.9 [16]. The KMO value observed in this study (0.87) was higher as compared to the KMO values of Italian (0.82), and Brazilian (0.81) validation studies [24, 25]. The suitability of data for EFA was also demonstrated using multivariate normality, factorability and multicollinearity analysis.

EFA was conducted for 11 COST items using parallel analysis for determining the number of factors to retain. Parallel analysis has already been discussed in results section and one factor with eigenvalue of 6.21 explaining 56.5% of the variance by non-rotated solution was identified. This result was consistent with the original COST development study which clearly identified one factor with eigenvalues of 5.68 explaining 89% of the variance on scree plot during factorability of the items [16]. COST validation studies conducted in Italy and Brazil reported a two-factor model; however, one factor model best represents the COST measure, and that factor was designated as FT [16, 24, 25]. The %age variance explained was lower in this study as compared to the parent COST study (89%) and Italian version (63%) but higher than the Brazilian version (51.6%) [16, 24, 25].

EFA showed that all the factor loadings in one factor model were ˃0.3 (range 0.35–0.97). Six items showed excellent loadings for Factor 1, one item with very good loading, one with good loading and two items with acceptable loadings (˃0.3) for Factor-1. These factor analysis loadings indicate the underlying construct can be considered as one factor domain as intended by the original study [16]. However, Chi-square goodness of fit test revealed the one factor model did not adequately depict the data mostly due to lower sample size but the results were acceptable and supporting the original COST development study as compared to Brazilian and Italian studies which reported two factor models.

The total COST score in this study indicated 62.1% patients suffered moderate impact, 20.7% mild and 6.9% experienced high impact FT. 69% of the patients responded not having money in savings or retirement to cover cancer care expenses and 72.4% felt that their illness has been a financial hardship to them and their family as well. Two studies conducted in Indian cancer patients revealed 76.5% and 84% cancer patients and households experienced catastrophic OOP expenditures [12, 34].

About 90% of the patients talked to attending oncologists regarding their financial hardships. Most patients were given information regarding financial assistance provided by Pradhan Mantri Jan Arogya Yojana and chief ministers medical assistance fund (Bihar) scheme both of which provide financial assistance to below poverty line households based on SECC-2011 data and 86% patients in this study received one or other form of financial assistance for treatment related costs. Due to cancer care, 62% of patients stopped working and 20% reported reduction in work. Despite financial assistance, high OOP expenses in diagnostics, medications for RT side effects, travelling, food, lodging and loss of income forced patients to employ coping strategies to pay for cancer care. 7% skipped follow-ups, 55% used their savings, 31% borrowed money on interest and 69% borrowed money from social nets. Rajpal *et al* [13] reported 40% of cancer patients use borrowings, sale of assets as coping strategies to pay for cancer treatment. As all the patients belonged to rural areas, majority complained that travelling from rural areas to radiotherapy centre, food and lodging expenses were high and difficult to pay adding to financial burden. A study conducted at AIIMS, New Delhi reported that about 59% of the cancer care was spent on transportation, food and lodging during RT treatment [14].

To the authors’ knowledge, this is the first study assessing the FT from the perspective of cancer patients in Indian health care system. FT and associated cost coping behaviour can prove detrimental particularly to the patients from lower socio-economic status.

In this study, the COST measure has demonstrated reliability and validity among head and neck cancer patients undergoing RT. However, the COST measure can prove to be a useful tool in measuring FT among other cancer subsites and treatment modalities including chemotherapy, surgery or multimodality treatments. COST measure will be useful in screening the patients at risk of FT and help in making better strategies and policies to alleviate the financial burden among cancer patients. Further studies are warranted for assessment of prevalence of FT among different cancer subsites in various cancer centres across India.

The limitation in this study is the small sample size. Based on the results of this study, we have initiated a prospective study of a larger sample size including other cancer subsites (breast cancer, abdominal cancer and other cancer patients) to further test the validation of COST for measuring FT and its association with HRQOL and other sociodemographic and clinical characteristics in cancer patients.

## Conclusion

In conclusion, this pilot study demonstrated an excellent reliability and validity of COST for measuring FT among head and neck radiation oncology patients. Further studies are warranted in different cancer subsites across various cancer centres in India. The clinical implications of FT and feasibility of the COST measure among other cancer subsites and treatment modalities need to be evaluated.

## Authors’ contributions

Mukhtar Ahmad Dar conceptualised and designed the study and contributed to literature search, data collection, data analysis and drafted the original manuscript. Richa Chauhan was study co-supervisor and contributed to patient recruitment, data collection, manuscript writing and resources necessary for this study. Krishna Kumar Sharma was the study supervisor and contributed in data analysis, review and editing the manuscript. Vinita Trivedi contributed to patient recruitment and manuscript editing and revisions. Sameer Dhingra and Krishna Murti have contributed to manuscript revisions, editing and proof-reading the final revised manuscript. The final version of the manuscript has been read and approved by all the authors for submission.

## Conflicts of interest

The authors declare that they have no conflicts of interest.

## Funding

This research did not receive any specific grant from funding agencies in the public, commercial or not for profit sectors.

## Figures and Tables

**Table 1.** Sociodemographic and clinical characteristics of study participants (*N* = 29).

Characteristics	*N* (%)	Characteristics	*N* (%)
Sex		Primary cancer site	
Male	24 (82.8)	Tongue	5 (17.2)
Female	5 (17.2)	Gingival buccal sulcus	5 (17.2)
Age group		Buccal mucosa	4 (13.7)
less than 50	14 (48.3)	Supraglottic larynx	3 (10.30
50–59	4 (13.8)	Hypopharynx	2 (6.9)
60 and above	11 (37.9)	Parotid	2 (6.9)
Education level		Thyroid	1 (3.5)
Not educated	10 (34.5)	Floor of mouth	1 (3.5)
Primary level	14 (48.3)	Para nasal sinus	1 (3.5)
Secondary level	3 (10.3)	Central arch lower alveolus	1 (3.5)
Graduate and above	2 (6.9)	Oropharynx	1 (3.5)
Occupation		Rt. tonsil	1 (3.5)
Employed	4 (13.8)	Sub mandibular gland	1 (3.5)
Unemployed	3 (10.3)	Lower lip	1 (3.5)
Labour work	10 (34.3)	Disease extent	
Farming	3 (10.3)	Metastatic	1 (3.5)
Homemaker	2 (6.9)	Non-metastatic	28 (96.5)
Others	7 (24.1)	Treatment modality	
Household size		Radiotherapy (RT)	5 (17.2)
1–5 members	12 (41.4)	RT + surgery (S)	14 (48.3)
6–10 members	15 (51.7)	RT + chemotherapy (CT)	6 (20.7)
More than 10 members	2 (6.9)	RT + CT + S	4 (13.8)
Marital status		Treatment intent	
Married	28 (96.5)	Definitive	15 (51.7)
Unmarried	1 (3.5)	Adjuvant	11 (38.0)
Annual household income (Rs.)		Palliative	3 (10.3)
≤50,000	17 (58.6)	Time since RT	
50,000–100,000	11 (37.9)	6 months or less	7 (24.1)
100,000–200,000	1 (3.5)	6 months to 12 months	10 (34.5)
Residence		12 months to 24 months	6 (20.7)
Rural	29 (100)	More than 24 months	6 (20.7)
Urban	0	Health insurance	
Current employment status		Yes	2 (6.9)
Stopped working	18 (62.1)	No	27 (93.1)
No change in work	5 (17.2)		
Reduction in working hours	6 (20.7)		

**Table 2.** Descriptive and reliability indices for 11 items of COST measure.

COST items	Mean	Std. deviation	Cronbach's alpha if item deleted
FT1	0.38	0.62	0.917
FT2	0.69	0.66	0.926
FT3	1.0	1.22	0.906
FT4	0.76	0.95	0.927
FT5	1.66	1.47	0.919
FT6	0.69	1.11	0.909
FT7	1.52	1.40	0.917
FT8	1.34	1.49	0.903
FT9	1.17	1.49	0.906
FT10	0.93	1.03	0.908
FT11	0.66	1.01	0.904

**Table 3.** Spearman’s correlation for 11 item COST measure (*N* = 29).

COST items		FT1	FT2	FT3	FT4	FT5	FT6	FT7	FT8	FT9	FT10	FT11
FT1	*r*_s_		0.31	0.68^a^	0.17	0.25	0.69^b^	0.47^a^	0.61^a^	0.38^b^	0.52^a^	0.69^a^
	*p*		0.10	0.00	0.38	0.19	0.00	0.01	0.00	0.04	0.00	0.00
FT2	*r*_s_	0.31		0.41^b^	0.30	0·13	0.27	0.17	0.31	0.10	0.27	0.37
	*p*	0.10		0.03	0.11	0.52	0.16	0·38	0.10	0.60	0.16	0.05
FT3	*r*_s_	0.68^a^	0.41^b^		0.28	0.52^a^	0.87^a^	0.59^a^	0.79^a^	0.57^a^	0.58^a^	0.78^a^
	*p*	0.00	0.03		0·15	0.00	0.00	0.00	0.00	0.00	0.00	0.00
FT4	*r*_s_	0.17	0.30	0.28		0.27	0.20	0.00	0.17	0.13	0.27	0.25
	*p*	0.38	0.11	0.15		0.16	0.29	1.00	0.39	0.50	0.15	0.20
FT5	*r*_s_	0.25	0.13	0.52^a^	0.27		0.45^b^	0.34	0.56^a^	0.79^a^	0.53^a^	0.51^a^
	*p*	0.19	0.52	0.00	0.16		0.01	0.07	0.00	0.00	0.00	0.00
FT6	*r*_s_	0.69^b^	0.27	0.87^a^	0.20	0.45^b^		0.62^a^	0.69^a^	0.54^a^	0.58^a^	0.75^a^
	*p*	0.00	0.16	0.00	0.29	0.01		0.00	0.00	0.00	0.00	0.00
FT7	*r*_s_	0.47^a^	0.17	0.59^a^	0.00	0.34	0.62^a^		0.64^a^	0.59^a^	0.51^a^	0.62^a^
	*p*	0.01	0.38	0.00	1.00	0.07	0.00		0.00	0.00	0.01	0.00
FT8	*r*_s_	0.61^a^	0.31	0.79^a^	0.17	0.56^a^	0.69^a^	0.643^a^		0.77^a^	0.71^a^	0.87^a^
	*p*	0.00	0.10	0.00	0.39	0.00	0.00	0.00		0.00	0.00	0.00
FT9	*r*_s_	0.38^b^	0.10	0.57^a^	0.13	0.79^a^	0.54^a^	0.59^a^	0.77^a^		0.71^a^	0.69^a^
	*p*	0.04	0.60	0.00	0.50	0.00	0·00	0.00	0.00		0.00	0.00
FT10	*r*_s_	0.52^a^	0.27	0.58^a^	0.27	0.53^a^	0.58^a^	0.58^a^	0.71^a^	0.71^a^		0.83^a^
	*p*	0.00	0.16	0.00	0.15	0.00	0.00	0.01	0.00	0.00		0.00
FT11	*r*_s_	0.69^a^	0.37	0.78^a^	0.25	0.51^a^	0.75^a^	0.75^a^	0.87^a^	0.69^a^	0.83^a^	
	*p*	0.00	0.05	0.00	0.20	0.00	0.00	0.00	0.00	0.00	0.00	

a Correlation is significant at the 0·01 level (2-tailed)

b Correlation is significant at the 0·05 level (2-tailed)

Spearman’s rho (*r*_s_) values are reported

**Table 4.** KMO and Bartlett's test (*N* = 29).

KMO Measure of sampling adequacy		0.865
Bartlett's test of sphericity	Approx. χ2	254.04
	*df*	55
	Sig. (*p*)	0.000^a^

a *p*-value significance level ˂0.001

**Table 5.** Factor loadings of 11 COST items from EFA (*N* = 29).

COST items	Factor loading 1
FT1 (I know that I have enough money in savings, retirement or assets to cover the costs of my treatment)	0.74
FT2 (My OOP medical expenses are more than I thought they would be)	0.37
FT3 (I worry about the financial problems I will have in the future as a result of my illness or treatment)	0.86
FT4 (I feel I have no choice about the amount of money I spend on care)	0.35
FT5 (I am frustrated that I cannot work or contribute as much as I usually do)	0.59
FT6 (I am satisfied with my current financial situation)	0.82
FT7 (I am able to meet my monthly expenses)	0.69
FT8 (I feel financially stressed)	0.92
FT9 (I am concerned about keeping my job and income, including work at home)	0.80
FT10 (My cancer or treatment has reduced my satisfaction with my present financial situation)	0.87
FT11 (I feel in control of my financial situation)	0.97

**Table 6.** Cost communication behaviour and cost coping strategies (*N* = 29).

Cost discussion with radiation oncologist	*n* (%)
Yes	26 (89·6%)
No	3 (10·4%)
Cost coping strategies employed	
Applied for financial assistance	25 (86%)
Used savings	16 (55%)
Borrowed from social nets	20 (69%)
Borrowed money on interest	9 (31%)
Sold assets	6 (21%)
Skipped follow-up or recommended treatment/test	2 (7%)
